# Health Risk Assessment of Heavy Metals Exposure from the Consumption of Cephalopods and Crustaceans in Peninsular Malaysia

**DOI:** 10.3390/toxics14030199

**Published:** 2026-02-27

**Authors:** Wan Nurul Farah Wan Azmi, Nurul Izzah Ahmad, Noraishah Mohammad Sham, Suraiami Mustar

**Affiliations:** 1 Environmental Health Research Centre, Institute for Medical Research, National Institutes of Health, Ministry of Health Malaysia, No. 1, Jalan Setia Murni U13/52, Seksyen U13, Setia Alam, Shah Alam 40170, Selangor, Malaysia; nizzah.a@moh.gov.my (N.I.A.); noraishah.ms@moh.gov.my (N.M.S.); 2 Nutrition, Metabolism and Cardiovascular Research Centre, Institute for Medical Research, National Institutes of Health, Ministry of Health Malaysia, No. 1, Jalan Setia Murni U13/52, Seksyen U13, Setia Alam, Shah Alam 40170, Selangor, Malaysia; suraiami@moh.gov.my

**Keywords:** heavy metals, seafood, squid, octopus, prawn, shrimp, risk assessment

## Abstract

Cephalopods and crustaceans are known to bioaccumulate heavy metals, potentially posing both non-carcinogenic and carcinogenic health risks to consumers. This study was conducted to determine heavy metal concentrations and assess associated health risks in the edible tissues of 84 cephalopod and crustacean samples. Heavy metal concentrations and assess associated health risks in the edible tissues of 84 cephalopod and crustacean samples collected from selected wholesale markets and major fish landing ports throughout Peninsular Malaysia. The analysis focused on nine heavy metals: selenium (Se), cadmium (Cd), lead (Pb), copper (Cu), zinc (Zn), antimony (Sb), tin (Sn), chromium (Cr), and manganese (Mn). The samples were digested using a microwave digestion system, and heavy metal concentrations were analysed using Inductively Coupled Plasma Mass Spectrometry (ICP-MS). Results showed that Mn was the most abundant metal, followed by Cr and Zn. Octopus (*C. indicus*) had the highest Mn concentration (5.01 mg/kg WW), while Rainbow shrimp (*P. sculptilis*) had the highest overall metal concentration (91.02 mg/kg WW). Significant differences were observed between cephalopods and crustaceans, with Cd and Sn concentrations being notably higher in cephalopods (*p* < 0.001). However, no significant associations were observed between heavy metal concentrations and sample weight or length, indicating a greater influence of environmental factors. Principal Component Analysis (PCA) explained 80.4% of the variance, with Cd, Sn, Pb, Cu, Zn, Cr, and Mn accounting for the majority of the variance. Estimated weekly intake (EWI) values ranged from 0.002 to 26.30 µg/kg bw/week for cephalopods and 8.02 × 10^−6^ to 243.175 µg/kg bw/week for crustaceans. All metal levels were below the permissible limits set by the Food and Agriculture Organisation of the United Nations/World Health Organisation (FAO/WHO). Hazard Index values were <1, indicating low non-carcinogenic risk, and Total Carcinogenic Risk values for Pb and Cr were below 1 × 10^−4^, suggesting negligible carcinogenic risk.

## 1. Introduction

Squid, cuttlefish, and octopus are examples of cephalopods, while prawns and shrimp are examples of crustaceans preferred for their rich taste, nutritional value, and benefits [1]. They are a popular food item in Malaysia, especially among adolescents. Malaysian adolescents consumed 84.9 ± 104.1 g of fish and seafood per day, with a higher amount contributed by the consumption of cephalopods and crustaceans at 80 ± 90 g/day and 63 ± 65 g/day, respectively [2]. Both seafood groups contributed a high amount of polyunsaturated fatty acids per day to the adolescents, with cephalopods (455.32 g/day) contributing more than twice as much as crustaceans (196.43 g/day) [3].

Food trace elements originate from both anthropogenic and natural events [4,5]. Metals are categorised into two groups: biologically vital and non-vital heavy metals. The essential group is required in small quantities for normal metabolic functions, but excessive intake can lead to adverse effects. In contrast, non-essential heavy metals can be toxic even at very low concentrations [4]. These widespread contaminants are readily absorbed by aquatic organisms, posing a significant risk to seafood consumers [6], as their toxicity is influenced by both dose and duration of exposure [4]. With the rising occurrence of hazardous substances in seafood, public awareness of food safety has increased, emphasising the need to safeguard health and prevent heavy metal–related food poisoning [6].

Research on heavy metals in cephalopods and crustaceans from Peninsular Malaysia was limited. However, it is possible to infer the widespread occurrence of certain heavy metals in both seafood species across various sampling sites, consistent with earlier research. No substantial differences in mercury levels were observed between cephalopods and crustaceans from different regions, and levels remained within World Health Organisation (WHO) guidelines [7]. Recent measurements of methylmercury show a mean of 0.0405 mg/kg wet weight in cephalopods and 0.0356 mg/kg in crustaceans [7]. An assessment of mature squid tissues from Kedah and Perlis in Peninsular Malaysia revealed that zinc (Zn) and copper (Cu) levels were below Food and Agriculture Organisation of the United Nations (FAO) 1985 limits, while cadmium (Cd) and lead (Pb) exceeded permissible thresholds [8]. Beyond cephalopods and crustaceans, heavy metal levels in Malaysian clams varied by species and sampling site [9].

Given the wide range of consumer preferences for seafood, weight and size are key considerations, as larger specimens often possess denser, chewier textures that enhance consumer satisfaction. Understanding the relationship between bioaccumulation and organism-specific traits, such as weight and size, may help reveal the physiological and biological factors that influence metal uptake [4]. However, previous studies have reported inconsistent patterns: while some species show a positive correlation between metal concentrations and organism size or weight, others demonstrate weak or even negative associations [7].

Although several studies have reported heavy metal contamination in seafood, comprehensive, systematic assessments of crustaceans and cephalopods across Peninsular Malaysia remain limited. The absence of spatially representative data restricts accurate evaluation of regional contamination patterns and associated human health risks, underscoring a critical knowledge gap that this study aims to address. Therefore, this study was conducted to determine the concentrations of heavy metals and to assess the associated health risks in the edible tissues of 84 samples (39 cephalopods and 45 crustaceans) collected from fish landing ports and wholesale markets across Peninsular Malaysia. The Estimated Weekly Intake (EWI) for each element was calculated to evaluate the potential risk from consuming contaminated seafood. In addition, non-carcinogenic risk was assessed by comparing the estimated exposure dose with the reference dose established by the USEPA. The analysis was further extended to include the Hazard Quotient (HQ) and Hazard Index (HI) for each metal, as well as the Target Cancer Risk (TCR) to estimate the incremental lifetime likelihood of developing cancer from exposure to probable carcinogens. The Principal Component Analysis (PCA) analysis was also conducted to provide a simplified visualisation and interpretation of multidimensional data and to explore the relationships among elements.

## 2. Materials and Methods

### 2.1. Sampling and Sample Preparation

**Figure 1:**
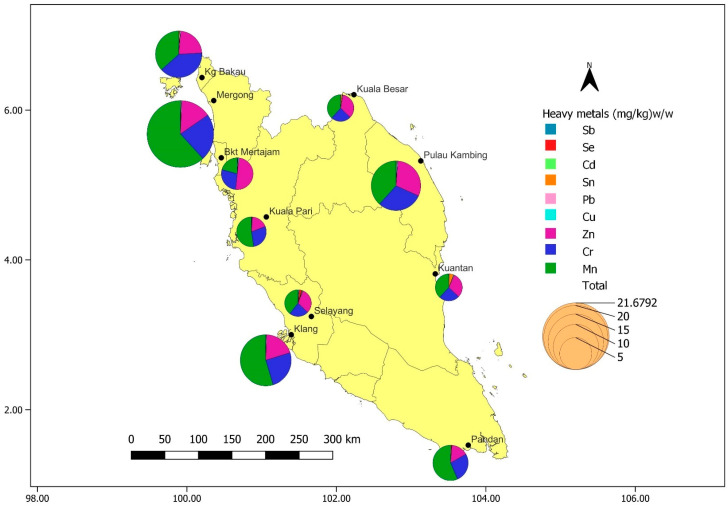
Distribution of heavy metals in cephalopods and crustaceans collected from sampling locations throughout Peninsular Malaysia.

Squids, cuttlefish, octopus (cephalopods), as well as prawn and shrimp (crustaceans) samples, were acquired from retailers’ markets and fishery landing sites in Peninsular Malaysia managed by the Malaysian authorities (Fisheries Development Authority of Malaysia). The study sites of the main fish and other seafood landing port distribution centres throughout Peninsular Malaysia are shown in Figure 1. The sampling, estimation, and data collection process follows the detailed technique disclosed by Ahmad et al. [7] throughout the seven-month sampling period from June to December 2009.

### 2.2. Equipment, Appliances and Reagents

Decontamination of all laboratory appliances was performed before analysis by immersing them in a 10% (*v*/*v*) nitric acid solution using 18.2 M-ohm water for an entire day. A mixture of indium (In), yttrium (Y), holmium (Ho), and scandium (Sc) as internal standard and isolated element solutions of nine elements, inclusive of antimony (Sb), cadmium (Cd), chromium (Cr), copper (Cu), plumbum (Pb), manganese (Mn), selenium (Se), tin (Sn), and zinc (Zn) at 10 mg/L were analysed for purity and concentration using Perkin Elmer^®^. Each stock solution (5 mL) was topped up to 50 mL with nitric acid (0.2%) in a volumetric flask to produce a 1 mg/L mixed working standard solution for calibration.

### 2.3. Process for Digesting Samples

A microwave (Multiwave 3000, Anton Paar, Austria) was used to digest a mixture of dried cephalopod or crustacean (0.5 g each) with hydrogen peroxide and highly concentrated nitric acid at 2 mL and 5 mL, respectively. Three stages of the maceration process were carried out by supplying power at 600 W, subsequently ramping for a few moments and maintaining. The procedure was continued by elevating the power to up to 1400 W, ramping (5 min), and maintaining (10 min). Lastly, the process was held for 15 min at zero power. Samples were later filtered with a 0.45 µm syringe filter, and ultrapure water was added to a 25 mL level. To produce reagent blanks devoid of samples, an identical technique was employed [10].

### 2.4. Heavy Metals Analysis

Inductively coupled plasma mass spectrometry (ICP-MS) ELAN 9000 (Perkin Elmer, Sciex, Canada), equipped with a quartz nebuliser, an autosampler, a convective spray chamber, and nickel sampler and skimmer cones, was used to analyse the presence of metals in the samples. A mixture of the nine elements mentioned earlier at 1 mg/L each was serially diluted to 10–100 μg/L and combined with a mixture of internal standards comprising 20 μg/L each of scandium, yttrium, indium, and holmium to produce a calibration curve. Seven consolidated reagent blanks containing each element and HNO_3_ were used to determine the limit of detection (LOD). Triplicate samples were analysed to ensure accuracy, and the standard deviations were computed for LOD quantification. To compare with PTWI, the heavy metal levels were converted from dry to wet weight readings using the formula by Ahmad et al. [7].

### 2.5. Assurance of Quality

To confirm the method’s accuracy, a standard suspension containing a mixture of components at 20 µg/L was added to the samples. The amount or level of the components was measured before and after the administration of the standard solution. Quality control for this analysis was ensured by analysing established source materials (NIST SRM^®^ 1946-Lake Superior Fish Tissue, Gaithersburg, MD, USA).

### 2.6. Estimated Weekly Intake (EWI)

An established Provisional Tolerable Weekly Intake (PTWI) by the Joint FAO/WHO Expert Committee on Food Additives (JECFA) was utilised to determine the Estimated Weekly Intake (EWI) [11]. Among the established heavy metals, PTWI includes total Cd, Cu, Pb, Sn, and Zn [12,13]. In Malaysia, individuals with a mean weight of 60 kg consume an average of 22.9 g of crustaceans and 45.0 g of cephalopods per day [14,15]. The EWI is estimated by multiplying the metal content (mg/kg, wet weight) by the weekly consumption per body weight (kg).

(1) $$EW=\frac{concentration\;of\;metal\;\left(mg/kg\right)\;wet\;weight\times weekly\;consumption}{body\;weight\;(kg)}$$

### 2.7. Health Risk Assessment

#### 2.7.1. Non-Carcinogen Risk Assessment

Risk evaluation helps predict the likelihood that risks will have a detrimental effect on human health. Cephalopods and crustaceans are the sources of exposure in this investigation. Heavy metals such as Se (5 × 10^−3^ mg/kg/day), Cd (1 × 10^−3^ mg/kg/day), Zn (3 × 10^−1^ mg/kg/day), Sb (4 × 10^−4^ mg/kg/day), and Mn (1.4 × 10^−1^ mg/kg/day) are included in the oral reference doses (RfDs) developed by USEPA [16]. However, the remaining heavy metals investigated in this study do not have a defined reference dose according to the USEPA Risk Information System (IRIS). Malaysian consumption of cephalopods per day per person for adults was 45.0 g/day, and crustaceans was 22.9 g/day [3]. The USEPA [16] has developed a formula to determine the risk of non-carcinogenic elements [17,18]:

(2) $$HQ=\;\frac{EF\;\times \;ED\;\times \;SIR\;\times \;C}{RfD\;\times \;BW\;\times \;AT}\;\times \;10^{-3}$$

In which, HQ: Hazard Quotient of a specific chemical, EF: exposure frequency (350 days/year), ED: exposure period (30 years), SIR: cephalopod/crustacean amount of consumption per day per person (g/day), C: metal concentration (mg/kg wet weight) in cephalopod or crustacean tissues, RfD: oral recommended dosage (IRIS, USEPA), BW: median weight of an adult (60 kg), AT: mean duration exposed to non-carcinogenic elements (365 days/year × number of exposure years, assuming 30 years).

The risk index was calculated following the equation below [17,18]:

(3) $$HI={HQ}_{Sb}+{HQ}_{Se}+{HQ}_{Cd}+{HQ}_{Zn}+{HQ}_{Mn}$$

In which the HI is the hazard index, the target hazard quotients for different consumptions: HQ_Sb_ for antimony (Sb), HQ_Se_ for selenium (Se), HQ_Cd_ for cadmium (Cd), HQ_Zn_ for zinc (Zn), HQ_Mn_ for manganese (Mn). Possible non-carcinogen risk concerns associated with the metals under study are indicated by HQ and HI values > 1.

#### 2.7.2. Target Cancer Risk (TCR)

The incremental likelihood of an individual acquiring cancer throughout their lifespan exposure to a probable carcinogen was used to evaluate carcinogen risks. The TCR was estimated following the formula below:

(4) $$TCR=\;\frac{EF\;\times \;ED\;\times \;SIR\;\times \;C}{BW\;\times \;ATc}\;\times \;CSFo\;\times \;10^{-3}$$

According to USEPA [19], ATc is the carcinogens latency period (365 days/year for 70 years), C is the content of metal in cephalopods and crustaceans (mg/kg), FIR is the cephalopods and crustaceans’ consumption rate (g/day), and CSFo is the carcinogenic slope factor of the oral ingestion (mg/kg bw/day). The US EPA suggested CSFo limits for Cr and Pb at 0.5 and 0.0085 mg/kg/day, respectively.

### 2.8. Data Distribution and Statistical Analysis

All data were examined, and inconsistencies were identified and corrected before being applied to the statistical analysis. Skewed or non-normal distribution data resulting from abnormalities or outliers will be analysed using a non-parametric technique. SPSS (version 2 for Windows, 2019) was used to calculate the median for each sample from the triplicate analysis, and the interquartile range was used to display the distribution of the data. Mann–Whitney’s and Kruskal–Wallis’s tests were employed to assess any significant difference between two groups and more than three groups, respectively. The *p*-value was established at *p* < 0.05. The association among heavy metals was analysed with principal component analysis (PCA). The distribution of heavy metal concentrations was analysed across various locations using QGIS software version 3.22.7. The dataset was interpolated to create a map highlighting the different heavy metal concentration levels at each location.

## 3. Results

### 3.1. Distribution of Heavy Metals in Peninsular Malaysia

Figure 1 illustrates the distribution of total metals in the cephalopods and crustaceans in the study locations. According to the findings, Mergong demonstrated the highest levels of total heavy metals, surpassing all other locations. The level of total heavy metals in Klang, Pulau Kambing, and Kg. Bakau is also substantial, even though relatively lower than Mergong. It is worth noting that the remaining locations exhibit nearly identical total heavy metal concentrations, with no significant differences. Kg. Bakau, Mergong, and Klang are located along the west coast of Peninsular Malaysia, adjacent to the Straits of Malacca. This region is subject to intense anthropogenic activities and receives substantial waste inputs from both land and sea-based sources. Primary potential sources of contamination include agricultural runoff, domestic effluents, industrial discharges, and shipping activities, all of which may contribute to elevated levels of metal pollution in the surrounding marine environment [20]. Meanwhile, Pulau Kambing, which is located near a petroleum refining plant, could contribute to elevated metal levels in that area [21].

### 3.2. Sample Characteristics and the Level of Heavy Metal in Cephalopods and Crustaceans

A total of eighty-four samples, including 39 cephalopods and 45 crustaceans, were analysed to determine the levels of Sb, Se, Cd, Sn, Pb, Cu, Zn, Cr, and Mn. There were eight species of cephalopods from three groups (cuttlefish, squid, and octopus) and 12 species of crustaceans from two groups (prawns and shrimps). Detailed weight and length ranges for each species are summarised in Table 1. Among the cephalopods, the lightest weighed approximately 10.0 g for Mitre (*L. chinensis*) and Sibogae (*L. sibogae*) squid, while the heaviest was 638.0 g, which also included Sibogae squid. The Western King prawn (*P. latisulcatus*), weighing around 270.0 g, was the heaviest crustacean, whereas the Indian white prawn (*P. indicus*) was the lightest at 3.6 g. Cephalopods vary in size from 12.0 to 61.2 cm, with the Sibogae squid being the largest at 61.2 cm and the Sword Tip squid (*L. edulis*) the smallest at 12.0 cm. The smallest crustacean, the Sand Velvet shrimp (*M. barbata*), measured 9.2 cm, while the largest, the Banana prawn (*M. merguiensis*), reached up to 48.1 cm.

The overall concentration of metals in cephalopods and crustaceans is shown in Table 2. The species showed a wide variety of metal values, as follows: Sb, 4.3 × 10^−4^–1.4 × 10^−3^; Se, not detected 6.9 × 10^−4^–2.5 × 10^−3^; Cd, 4.2 × 10^−4^–3.3 × 10^−3^; Sn, 1.1 × 10^−2^–6.5 × 10^−2^; Pb, 8.5 × 10^−4^–1.7 × 10^−2^; Cu, 1.0 × 10^−2^–5.3 × 10^−2^; Zn, 1.4 × 10^−1^–1.73; Cr, 4.6 × 10^−1^–2.52 and Mn, 1.1 × 10^−1^–5.01 mg/ kg wet weight for cephalopods. In crustaceans, Sb, 2.5 × 10^−4^–4.2 × 10^−3^; Se, 2.6 × 10^−4^–1.2 × 10^−2^; Cd, 6.0 × 10^−6^–1.5 × 10^−2^; Sn, 1.2 × 10^−4^–3.4 × 10^−1^; Pb, ND—1.0 × 10^−2^; Cu, ND—1.2 × 10^−1^; Zn, ND—9.53; Cr, ND—15.56; and Mn, ND—91.02 mg/ kg wet weight. These findings implied that different metals accumulated differentially in the marine species. The most prevalent metal in both taxa was Mn, followed by Cr and Zn. Among cephalopods, the octopus species (*C. indicus*) had the highest Mn content, measuring 5.01 mg/kg wet weight, followed by the squid species (*L. uyii*), with 1.27 mg/kg wet weight. Meanwhile, the maximum values of 91.02, 9.67, and 3.79 mg/kg wet weight were observed for crustacean shrimp groups (*P. sculptilis*, *P. hardwickii*, *M. affinis*). Cr concentration was highest in cephalopods in the octopus group (*C. indicus*: 2.52 mg/kg wet weight) and cuttlefish group (*L. uyii*: 1.17 mg/kg wet weight), as well as crustaceans in the shrimp group (*P. sculptilis*: 15.56 mg/kg and *P. hardwickii*: 4.08 mg/kg wet weight). The utmost Zn levels among cephalopods were exhibited by the octopus group (*C. indicus*: 1.73 mg/kg wet weight), and among crustaceans, by the shrimp group (*P. sculptilis*: 9.53 mg/kg wet weight). Due to the absence of Pb, Cu, Zn, Cr, and Mn, crustaceans in the prawn group (*P. japonicus*) had the lowest overall levels of heavy metals.

### 3.3. Comparison of Heavy Metal Concentration Between Cephalopods and Crustaceans

**Figure 2:**
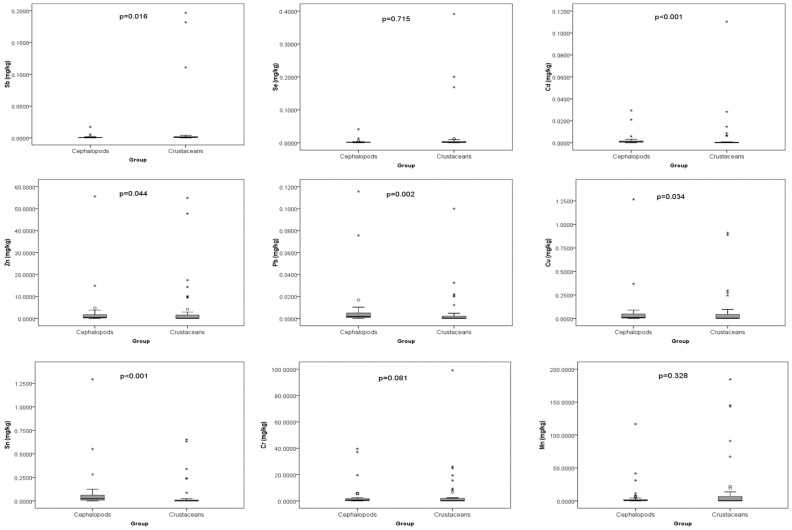
Comparison of heavy metal concentrations between cephalopods and crustaceans. * outliers.

Figure 2 summarises the overall analysis of heavy metal content in cephalopods and crustaceans. Significant variation was observed for Sb, Cd, Zn, Pb, Cu, and Sn between the two groups, with Cd and Sn being markedly higher in cephalopods (*p* < 0.001). Additionally, Pb, Sb, Cu, and Zn also differed significantly (*p* < 0.05) between cephalopods and crustaceans. However, other metals (Se, Cr, and Mn) were not found to be statistically significant, which may be attributed to the influence of outliers in the dataset.

### 3.4. Heavy Metal Relationships Between Weight and Length of the Samples

**Figure 3:**
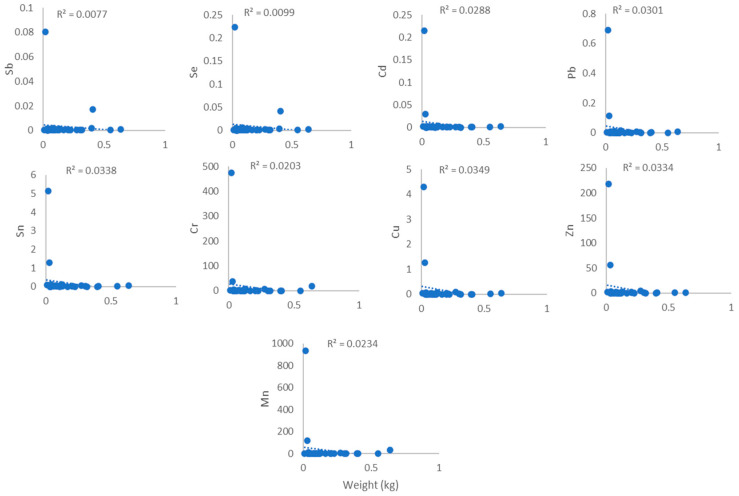
Relationship between heavy metal levels (mg/kg) and weight of cephalopod samples from Peninsular Malaysia.

**Figure 4:**
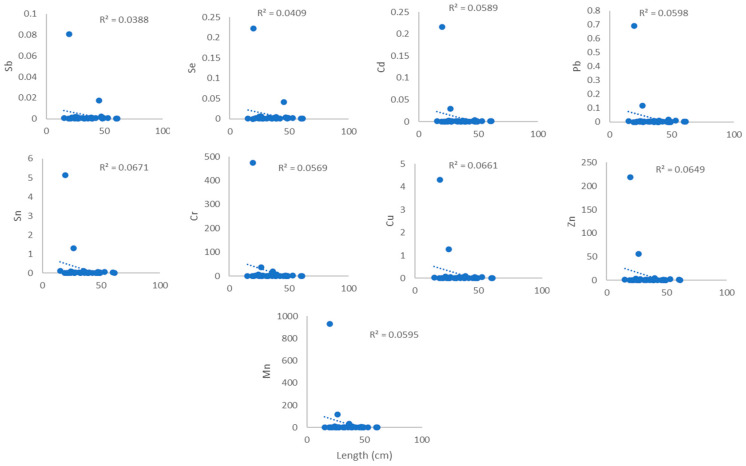
Relationship between heavy metal levels (mg/kg) and length of cephalopod samples from Peninsular Malaysia.

**Figure 5:**
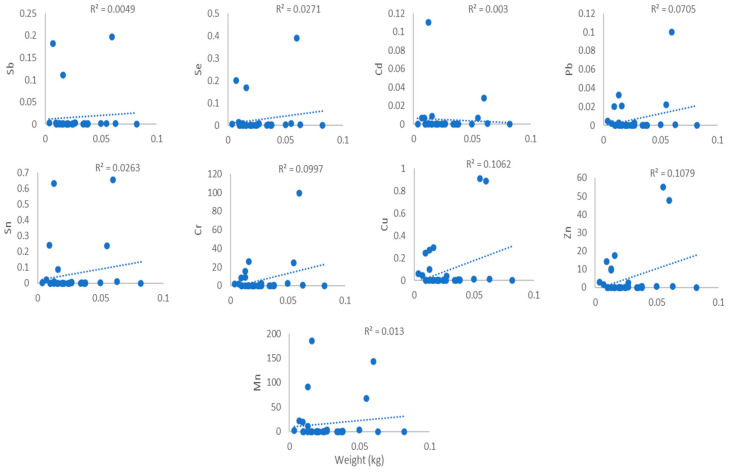
Relationship between heavy metal levels (mg/kg) and weight of crustacean samples from Peninsular Malaysia.

**Figure 6:**
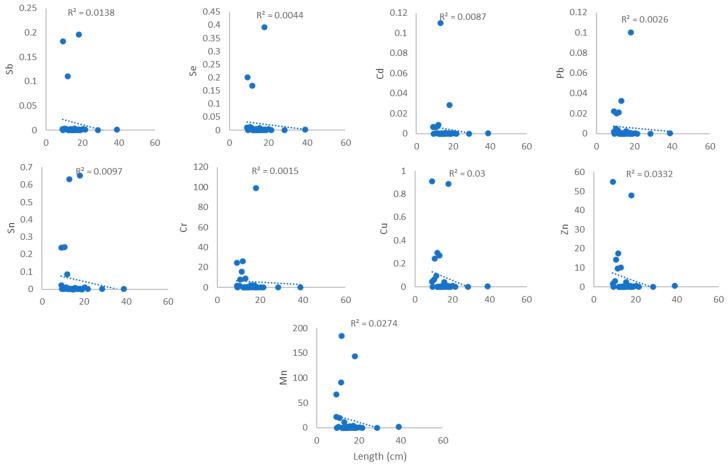
Relationship between heavy metal levels (mg/kg) and length of crustacean samples from Peninsular Malaysia.

Figure 3, Figure 4, Figure 5 and Figure 6 show the association between heavy metal levels and the weight and length of cephalopod and crustacean samples from Peninsular Malaysia. The minimum and maximum weights of cephalopods and crustaceans were 10.0 g and 638 g, and 3.6 g and 82.0 g, respectively, for the lengths: 12.0 cm, 61.2 cm (cephalopods), and 9.2 cm and 48.1 cm (crustaceans) (Table 1). Crustaceans, *P. merguiensis*, with an average length and weight of 18.77 cm and 44 g, show high levels of all heavy metals except for Cd. Penaeus indicus, with a length of 10.4 cm, exhibits high levels of Cd. A sample of *Loligo edulis* measuring 19.7 cm and weighing 20 g shows significantly elevated levels of all heavy metals. Results show that the sample’s morphology does not affect the quantity of metals in cephalopods and crustaceans. There was no discernible relation/connection between the levels of heavy metals in cephalopods and weight, with R^2^ values ranging from 0.0077 to 0.0349 (Figure 3). Similar results were observed for the connection/link between heavy metals and length, with R^2^ values from 0.0388 to 0.0671 (Figure 4). Similarly, crustaceans exhibited weak to negligible correlations: R^2^ values ranging from 0.003 to 0.1079 for weight (Figure 5) and R^2^ values from 0.0015 to 0.0332 for length (Figure 6).

### 3.5. Principal Component Analysis

**Figure 7:**
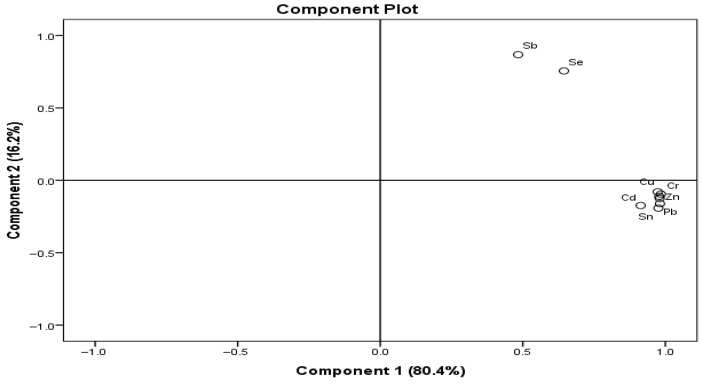
Rotation component loading plot of principal component analysis for heavy metals.

To explore the association among heavy metals in cephalopods and crustaceans, principal component analysis (PCA) was conducted. The component matrix percentages for PCA which shows that the first two components explained 96.6% of the cumulative contribution in the samples. Principal component 1 accounted for 80.4% of the total variance in the samples, with Cd (91.3%), Sn (97.6%), Pb (98.1%), Cu (98.0%), Zn (97.8%), Cr (98.5%), and Mn (97.3%) contributing most. Principal component 2 explained 16.2% of the total variance in the samples, with Sb accounting for 86.8% and Se accounting for 75.6% (Table S1). The PCA rotated component loading plot depicted in Figure 7 represents the relationship between nine metals. Sb and Se were associated with one another in the same component, while the other seven metals were clustered together. The close clustering of the seven metals in the PCA loading plot indicates similar contribution patterns across principal components, suggesting that these elements share familiar sources or are influenced by similar environmental and geochemical processes. Their closeness also implies comparable bioavailability and uptake pathways within the organisms. In contrast, Sb and Se are positioned farther from the main cluster, reflecting weaker correlations with the other metals and distinct loading behaviours. This separation indicates element-specific accumulation patterns, likely associated with differing contamination sources, geochemical properties, or biological regulation mechanisms [22]. Overall, the PCA results highlight the dominance of shared environmental influences for most metals, while Sb and Se exhibit more independent behaviour within the multivariate structure.

### 3.6. Health Risk Assessment from Consumption of Cephalopods and Crustaceans

The potential health risks of consuming cephalopods and crustaceans among the Malaysian population were estimated using the weekly intake (EWI) as shown in Table 3. The values were correlated with the PTWI values proposed by the FAO/WHO with Cd (7 µg/kg bw/week), Pb (25 µg/kg bw/week), Cu (3500 µg/kg bw/week), Zn (7000 µg/kg bw/week), and Sn (14,000 µg/kg bw/week) The EWI of metals from the consumption of cephalopods and crustaceans shows distinct variation across species. The EWI for cephalopods was within the limits of 0.002 to 26.30 µg/kg bw/week, whereas crustaceans ranged from 8.02 × 10^−6^ to 243.175 µg/kg bw/week. All detected heavy metals in cephalopods and crustaceans were below the FAO/WHO established thresholds. Overall, *P. monodon* consistently had the lowest concentrations of most metals, while *P. japonicus*, *C. indicus*, and *P. sculptilis* stood out for having elevated levels across multiple elements.

**Figure 8:**
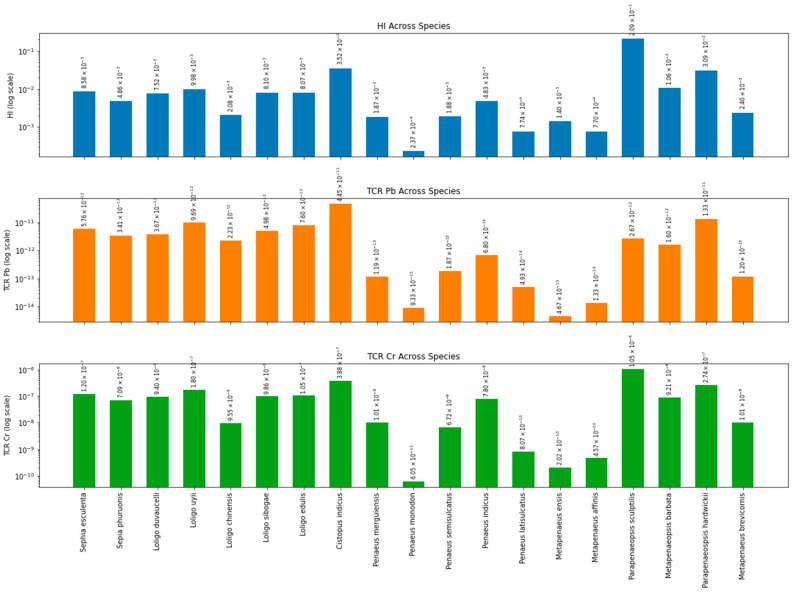
Health risk assessment from consumption of cephalopods and crustaceans in Peninsular Malaysia.

Figure 8 and Table S2 summarise estimates of the health risks of consuming cephalopods and crustaceans among adult Malaysians. Non-carcinogenic risk assessments include hazard quotients (HQ) and hazard index (HI), whereas for carcinogenic risks, the lifetime target cancer risk (TCR) was computed. The highest risk of heavy metals to health in both marine organisms is Mn, which ranged from 5.65 × 10^−4^ to 2.57 × 10^−2^ in cephalopods and up to 1.97 × 10^−1^ (where detectable) in crustaceans. The total hazards denoted as hazard index (HI) ranged from 2.08 × 10^−3^ to 3.52 × 10^−2^ for cephalopods and from non-detected to 2.09 × 10^−1^ for crustaceans. Values less than 1 (HI < 1) demonstrate the low hazard risk of non-carcinogens to consumers.

Carcinogenic risks were assessed for Pb and Cr, determined as the cumulative likelihood of a person acquiring cancer throughout their entire life after being exposed to that cancer-causing substance. The values for Pb in cephalopods were within the range of 2.23 × 10^−12^ to 4.45 × 10^−11^, and for crustaceans, much lower, from non-detectable to 1.33 × 10^−11^. Cr was slightly higher for both with values between 9.55 × 10^−9^ and 3.88 × 10^−7^ for cephalopods, and for crustaceans from non-detectable to 1.05 × 10^−6^. The unacceptable level for TCR was above 10^−4^, which was not found in any of the tested samples. Both seafoods showed TCR values within the range of 10^−6^ to 10^−4^ below the USEPA hazard limit, with only one crustacean species (*P. sculptilis*) showing a value above 10^−6^ (1.05 × 10^−6^), indicating negligible carcinogenic risk from consumption [23].

## 4. Discussion

### 4.1. Heavy Metal Levels

Three species of cephalopods (cuttlefish, squid and octopus) and two species of crustaceans (prawns and shrimp) were analysed for nine heavy metals: Sb, Se, Cd, Sn, Pb, Cu, Zn, Cr, and Mn. Ten sampling ports throughout Peninsular Malaysia: Pandan (Johor), Klang and Selayang (Selangor), Kuala Pari (Perak), Bukit Mertajam (Pulau Pinang), Mergong (Kedah), Kg Bakau (Perlis), Kuala Besar (Kelantan), Pulau Kambing (Terengganu), and Kuantan, (Pahang) were visited for sample collection (Figure 1). The heavy metal concentrations differed between cephalopod and crustacean species, with Mn, Cr, and Zn as the predominant elements, in the sequence Mn > Zn > Cr (Table 2). Metal uptake in aquatic organisms occurs through various pathways, including trophic (ingestion), dietary (ingestion), sediment (absorption from seawater and pore water), and maternal transfer [24]. The bioaccumulation rate depends on various factors, including environmental conditions (pH, salinity, water temperature, levels of metals in the surrounding environment, and duration of exposure) and biological factors (species, feeding habits, and growth stage) [4]. The digestive gland is the primary structure responsible for accumulating heavy metals in both taxa, though element distribution varies by different types of metals, namely Cd, Pb, Cu and Zn [1,25]. All detected metal concentrations remained within FAO safety limits [14], consistent with global studies indicating safe consumption levels. However, continuous monitoring of environmental factors contributing to metal bioaccumulation is essential to prevent future contamination risks [26].

### 4.2. Relationship Between the Heavy Metals and Weight and Length

Pearson’s method was utilised to assess the association between levels of heavy metals and the weight and length of cephalopods and crustaceans, as shown in Figure 3, Figure 4, Figure 5 and Figure 6. Results indicated no positive correlation among the tested parameters. These findings may suggest that factors such as habitat, exposure to pollutants, and diet have a greater influence on heavy metal concentrations than the physical properties. Inconsistent trends were observed in previous studies, with recent research on *Octopus vulgaris* (cephalopod) reporting that contamination levels in the region exert a greater effect on heavy metal absorption than morphological factors [27], while in some species, heavy metal concentrations increase with size and length due to cumulative exposure [28,29]. Previous studies indicate that heavy metal concentrations are often not significantly correlated with organism length or weight and do not differ markedly among tissues despite size variation [30]. Significant relationships are observed in only a few cases and vary by species and metal type, suggesting that body size is not a consistent predictor of metal accumulation. Instead, environmental exposure and physiological factors appear to play a more dominant role [31].

### 4.3. Evaluation of Health Risk Assessment

Cephalopods and crustaceans can be significant sources of heavy metals for seafood enthusiasts. Therefore, the EWI of total heavy metals in cephalopods and crustaceans among Malaysian adults was determined, and the results showed variation among species. The values were below the permitted thresholds for Cd, Pb, Cu, Zn, and Sn, indicating a low potential risk, consistent with earlier studies [23,32]. The HQ and lifetime TCR were also considered in the risk assessment. The HI values, presenting the total HQ, were also calculated. HQ values for non-carcinogenic risk factors for Sb, Se, Cd, Zn, and Mn in cephalopods and crustaceans were below 1, indicating that consumption is safe for consumers. Similarly, TCR for carcinogenic risk factors involving Pb and Cr remained below USEPA acceptable risk thresholds (10^−6^ to 10^−4^) [23], indicating negligible carcinogenic risk from consumption. These findings corroborate earlier studies on various commercial cephalopod and crustaceans from different sampling ports [33,34]. On the contrary, another study reported adverse carcinogenic health risks associated with seven crustacean species from two locations of the South China Sea [35]. This indicates that the health risks of consuming particular crustacean species may vary depending on their region or origin. Although consumption of both taxa appears safe for healthy adults, sensitive populations, including children, pregnant women, and the elderly, are more susceptible to adverse health effects from heavy metal exposure. Systematic evidence indicates that these groups experience greater health risks than the general adult population and therefore should consume lower amounts than healthy adults [36].

### 4.4. Limitation

Even though this study was conducted in 2009, the data presented remain valuable for future research examining trends in contaminant levels. This study was limited to Peninsular Malaysia. To address this limitation, a nationwide investigation initiated in 2023 is currently underway, encompassing both Peninsular and East Malaysia, aiming to provide a comprehensive, up-to-date assessment of contaminant levels across Malaysia.

## 5. Conclusions

This study measured nine heavy metals in 84 cephalopod and crustacean samples collected across Peninsular Malaysia. Mergong showed the highest overall metal levels, with Mn being the most abundant metal in both groups. Cephalopods had significantly higher Cd and Sn concentrations, while Rainbow shrimp (*P. sculptilis*) showed the highest single metal concentration. No significant associations were observed between heavy metal concentrations and sample weight or length, indicating that environmental factors (e.g., habitat and pollutant exposure) have a greater influence than physical attributes. All metal levels were below FAO/WHO limits, and both the Hazard Index and Total Carcinogenic Risk values indicated negligible health risks from consuming these seafoods in Peninsular Malaysia.

## Figures and Tables

**Table 1:** List of cephalopod and crustacean samples in this study.

Group/Commercial Name	Scientific Name	N	Weight Range (g)	Length Range (cm)
**Cephalopods**				
**Cuttlefish**				
Golden cuttlefish	*Sephia esculenta*	6	46.0–396.0	24.8–47.0
Pharaoh cuttlefish	*Sepia pharaonis*	1	190.0–550.0	31.2–49.2
	**Total**	**7**		
**Squid**				
Indian squid	*Loligo duvaucelii*	10	46.0–406.0	16.0–53.0
Little squid	*Loligo uyii*	1	39.0–72.0	24.0–38.4
Mitre squid	*Loligo chinensis*	6	10.0–302.0	30.6–60.0
Sibogae squid	*Loligo sibogae*	6	10.0–638.0	21.7–61.2
Sword tip squid	*Loligo edulis*	8	20.0–64.0	12.0–27.6
	**Total**	**31**		
**Octopus**				
Old women octopus	*Cistopus indicus*	1	132.0	47.2
	**All cephalopods**	**39**		
**Crustaceans**				
**Prawn**				
Banana prawn	*Penaeus merguiensis*	5	20.0–63.0	10.0–48.1
Giant tiger prawn	*Penaeus monodon*	1	20.0–42.0	28.6–31.2
Green tiger prawn	*Penaeus semisulcatus*	2	25.0–26.8	13.8–14.9
Indian white prawn	*Penaeus indicus*	8	3.6–50.0	9.3–18.6
Kuruma prawn	*Penaeus japonicus*	1	50.0	19.4
Western king prawn	*Penaeus latisulcatus*	5	10.0–270.0	13.3–21.7
	**Total**	**22**		
**Shrimp**				
Greasyback shrimp	*Metapenaeus ensis*	3	20.0–60.0	13.3–18.1
Pink shrimp	*Metapenaeus affinis*	4	16.0–20.0	10.0–16.4
Rainbow shrimp	*Parapenaeopsis sculptilis*	3	13.0–19.0	11.5–14.3
Sand velvet shrimp	*Metapenaeopsis barbata*	7	5.0–55.0	9.2–15.0
Spear shrimp	*Parapenaeospsis hardwickii*	2	9.4–38.0	10.7–27.1
Yellow shrimp	*Metapenaeus brevicornis*	4	10.0–82.0	9.5–39.0
	**Total**	**23**		
	**All crustaceans**	**45**		

**Table 2:** Heavy metal concentrations in cephalopods and crustaceans in Peninsular Malaysia.

Group/ Commercial Name	Scientific Name	N	Heavy Metals Concentrations in Median, mg/kg Wet Weight (IQR)								
			Sb	Se	Cd	Sn	Pb	Cu	Zn	Cr	Mn
**Cephalopods**											
Cuttlefish											
Golden cuttlefish	*Sephia esculenta*	6	1.2 × 10^−3^ (1.1 × 10^−3^)	2.5 × 10^−3^ (3.4 × 10^−3^)	7.6 × 10^−4^ (1.4 × 10^−3^)	3.1 × 10^−2^ (7.1 × 10^−2^)	2.2 × 10^−3^ (4.9 × 10^−3^)	1.5 × 10^−2^ (4.8 × 10^−2^)	6.3 × 10^−1^ (1.87)	7.8 × 10^−1^ (2.39)	7.8 × 10^−1^ (2.48)
Pharaoh cuttlefish	*Sepia phuruonis*	1	6.0 × 10^−4^	1.6 × 10^−3^	7.2 × 10^−4^	2.4 × 10^−2^	1.3 × 10^−3^	1.2 × 10^−2^	4.5 × 10^−1^	4.6 × 10^−1^	3.8 × 10^−1^
	**Total**	**7**									
Squid											
Indian squid	*Loligo duvaucelli*	10	5.8 × 10^−4^ (5.7 × 10^−4^)	1.5 × 10^−3^ (1.3 × 10^−3^)	7.0 × 10^−4^ (8.1 × 10^−4^)	1.3 × 10^−2^ (1.5 × 10^−2^)	1.4 × 10^−3^ (1.7 × 10^−3^)	1.0 × 10^−2^ (2.0 × 10^−2^)	4.5 × 10^−1^ (9.1 × 10^−1^)	6.1 × 10^−1^ (1.40)	9.1 × 10^−1^ (1.57)
Little squid	*Loligo uyii*	1	6.8 × 10^−4^	1.5 × 10^−3^	1.4 × 10^−3^	4.9 × 10^−2^	3.7 × 10^−3^	1.2 × 10^−2^	4.2 × 10^−1^	1.17	1.27
Mitre squid	*Loligo chinensis*	6	4.3 × 10^−4^ (4.1 × 10^−4^)	6.9 × 10^−4^ (2.3 × 10^−3^)	4.2 × 10^−4^ (1.0 × 10^−3^)	1.1 × 10^−2^ (3.4 × 10^−2^)	8.5 × 10^−4^ (5.5 × 10^−3^)	3.9 × 10^−3^ (5.3 × 10^−2^)	1.4 × 10^−1^ (2.74)	6.2 × 10^−2^ (3.08)	1.1 × 10^−1^ (3.57)
Sibogae squid	*Loligo sibogae*	6	5.1 × 10^−4^ (3.4 × 10^−4^)	1.3 × 10^−3^ (1.7 × 10^−3^)	1.4 × 10^−3^ (2.2 × 10^−3^)	4.7 × 10^−2^ (1.2 × 10^−1^)	1.9 × 10^−3^ (4.5 × 10^−3^)	1.3 × 10^−2^ (3.2 × 10^−2^)	5.9 × 10^−1^ (1.30)	6.4 × 10^−1^ (5.49)	8.9 × 10^−1^ (9.10)
Sword tip squid	*Loligo edulis*	8	5.7 × 10^−4^ (3.3 × 10^−3^)	1.2 × 10^−3^ (9.2 × 10^−3^)	6.8 × 10^−4^ (2.5 × 10^−2^)	1.6 × 10^−2^ (9.2 × 10^−1^)	2.9 × 10^−3^ (9.6 × 10^−2^)	2.7 × 10^−2^ 8.2 × 10^−1^)	9.7 × 10^−1^ (35.2)	6.8 × 10^−1^ (38.3)	7.9 × 10^−1^ (79.2)
	**Total**	**31**									
Octopus											
Old women octopus	*Cistopus indicus*	1	1.4 × 10^−3^	3.1 × 10^−3^	3.3 × 10^−3^	6.5 × 10^−2^	1.7 × 10^−2^	5.3 × 10^−2^	1.73	2.52	5.01
	**All cephalopods**	**39**									
**Crustaceans**											
Prawn											
Banana prawn	*Penaeus merguiensis*	5	1.2 × 10^−3^ (1.3 × 10^−3^)	1.8 × 10^−3^ (3.1 × 10^−3^)	3.8 × 10^−4^ (4.6 × 10^−4^)	5.4 × 10^−3^ (8.7 × 10^−3^)	8.9 × 10^−5^ (2.9 × 10^−4^)	1.3 × 10^−3^ (5.4 × 10^−3^)	4.7 × 10^−2^ (2.3 × 10^−1^)	1.5 × 10^−1^ (1.6 × 10^−1^)	1.7 × 10^−1^ (1.1 × 10^−1^)
Giant tiger prawn	*Penaeus monodon*	1	2.5 × 10^−4^	2.6 × 10^−4^	6.0 × 10^−6^	8.4 × 10^−5^	7.0 × 10^−6^	8.2 × 10^−5^	3.4 × 10^−3^	9.0 × 10^−4^	1.3 × 10^−3^
Green tiger prawn	*Penaeus semisulcatus*	2	1.3 × 10^−3^	2.5 × 10^−3^	9.3 × 10^−5^	1.4 × 10^−3^	1.4 × 10^−4^	1.0 × 10^−3^	5.5 × 10^−2^	1.0 × 10^−1^	2.8 × 10^−1^
Indian white prawn	*Penaeus indicus*	8	1.2 × 10^−3^ (1.7 × 10^−3^)	1.8 × 10^−3^ (2.9 × 10^−3^)	1.3 × 10^−4^ (3.9 × 10^−4^)	1.6 × 10^−3^ (4.8 × 10^−3^)	5.1 × 10^−4^ (3.9 × 10^−3^)	7.8 × 10^−3^ (5.6 × 10^−2^)	3.7 × 10^−1^ (2.53)	1.16 (2.21)	1.53 (11.15)
Kuruma prawn	*Penaeus japonicus*	1	4.2 × 10^−3^	1.2 × 10^−2^	1.5 × 10^−2^	3.4 × 10^−1^	ND	ND	ND	ND	ND
Western king prawn	*Penaeus latisulcatus*	5	7.6 × 10^−4^ (1.9 × 10^−3^)	7.4 × 10^−4^ (4.1 × 10^−3^)	1.6 × 10^−5^ (4.7 × 10^−4^)	2.8 × 10^−4^ (3.9 × 10^−3^)	3.7 × 10^−5^ (1.2 × 10^−3^)	2.4 × 10^−4^ (2.1 × 10^−2^)	1.1 × 10^−2^ (1.25)	1.2 × 10^−2^ (1.18)	2.9 × 10^−2^ (1.55)
	**Total**	**22**									
Shrimp											
Greasyback shrimp	*Metapenaeus ensis*	3	1.5 × 10^−3^	2.1 × 10^−3^	4.6 × 10^−5^	1.2 × 10^−4^	3.5 × 10^−6^	2.5 × 10^−5^	1.1 × 10^−3^	3.0 × 10^−3^	4.6 × 10^−3^
Pink shrimp	*Metapenaeus affinis*	4	7.8 × 10^−4^ (1.4 × 10^−3^)	1.0 × 10^−3^ (2.1 × 10^−3^)	1.9 × 10^−5^ (1.0 × 10^−4^)	1.3 × 10^−4^ (1.7 × 10^−3^)	1.0 × 10^−5^ (3.7 × 10^−4^)	1.5 × 10^−4^ (9.7 × 10^−3^)	8.8 × 10^−3^ (5.1 × 10^−1^)	6.8 × 10^−3^ (1.36)	1.9 × 10^−2^ (6.08)
Rainbow shrimp	*Parapenaeopsis sculptilis*	3	2.1 × 10^−3^	6.1 × 10^−3^	4.6 × 10^−4^	1.0 × 10^−2^	2.0 × 10^−3^	7.8 × 10^−2^	9.53	15.56	91.02
Sand velvet shrimp	*Metapenaeopsis barbata*	7	1.4 × 10^−3^ (1.4 × 10^−3^)	2.8 × 10^−3^ (5.1 × 10^−3^)	4.4 × 10^−4^ (3.2 × 10^−2^)	1.9 × 10^−2^ (3.4 × 10^−1^)	1.2 × 10^−3^ (2.5 × 10^−2^)	1.6 × 10^−2^ (4.3 × 10^−1^)	9.3 × 10^−1^ (21.31)	1.37 (13.17)	3.79 (24.83)
Spear shrimp	*Parapenaeospsis hardwickii*	2	1.8 × 10^−3^	7.0 × 10^−3^	3.4 × 10^−3^	1.2 × 10^−1^	1.0 × 10^−2^	1.2 × 10^−1^	7.18	4.08	9.67
Yellow shrimp	*Metapenaeus brevicornis*	4	5.7 × 10^−4^ (8.3 × 10^−2^)	1.3 × 10^−3^ (1.3 × 10^−1^)	8.9 × 10^−5^ (6.6 × 10^−3^)	1.3 × 10^−3^ (6.5 × 10^−2^)	9.0 × 10^−5^ (1.6 × 10^−2^)	2.5 × 10^−3^ (2.2 × 10^−1^)	2.2 × 10^−1^ (13.2)	1.5 × 10^−1^ (19.6)	7.5 × 10^−1^ (138.8)
	**Total**	**23**									
	**All crustaceans**	**45**									

N: no. of samples. IQR: interquartile range. ND: not detected.

**Table 3:** Estimated weekly intake (EWI) from consumption of cephalopods and crustaceans by adults in Peninsular Malaysia (µg/kg b.wt/week).

Species	Sb	Se	Cd	Sn	Pb	Cu	Zn	Cr	Mn
*Sephia esculenta*	6.30 × 10^−3^	1.31 × 10^−2^	3.99 × 10^−3^	1.63 × 10^−1^	1.16 × 10^−2^	7.88 × 10^−2^	3.31	4.10	4.10
*Sepia phuruonis*	3.15 × 10^−3^	8.40 × 10^−3^	3.78 × 10^−3^	1.26 × 10^−1^	6.83 × 10^−3^	6.30 × 10^−2^	2.36	2.42	2.00
*Loligo duvaucelli*	3.05 × 10^−3^	7.88 × 10^−3^	3.68 × 10^−3^	6.83 × 10^−2^	7.35 × 10^−3^	5.25 × 10^−2^	2.36	3.20	4.78
*Loligo uyii*	3.57 × 10^−3^	7.88 × 10^−3^	7.35 × 10^−3^	2.57 × 10^−1^	1.94 × 10^−2^	6.30 × 10^−2^	2.21	6.14	6.67
*Loligo chinensis*	2.26 × 10^−3^	3.62 × 10^−3^	2.21 × 10^−3^	5.78 × 10^−2^	4.46 × 10^−3^	2.05 × 10^−2^	7.35 × 10^−1^	3.26 × 10^−1^	5.78 × 10^−1^
*Loligo sibogae*	2.68 × 10^−3^	6.83 × 10^−3^	7.35 × 10^−3^	2.47 × 10^−1^	9.98 × 10^−3^	6.83 × 10^−2^	3.10	3.36	4.67
*Loligo edulis*	2.99 × 10^−3^	6.30 × 10^−3^	3.57 × 10^−3^	8.40 × 10^−2^	1.52 × 10^−2^	1.42 × 10^−1^	5.09	3.57	4.15
*Cistopus indicus*	7.35 × 10^−3^	1.63 × 10^−2^	1.73 × 10^−2^	3.41 × 10^−1^	8.93 × 10^−2^	2.78 × 10^−1^	9.08	1.32 × 10^1^	2.63 × 10^1^
*Penaeus merguiensis*	3.21 × 10^−3^	4.81 × 10^−3^	1.02 × 10^−3^	1.44 × 10^−2^	2.38 × 10^−4^	3.47 × 10^−3^	1.26 × 10^−1^	4.01 × 10^−1^	4.54 × 10^−1^
*Penaeus monodon*	6.68 × 10^−4^	6.95 × 10^−4^	1.60 × 10^−5^	2.24 × 10^−4^	1.87 × 10^−5^	2.19 × 10^−4^	9.08 × 10^−3^	2.40 × 10^−3^	3.47 × 10^−3^
*Penaeus semisulcatus*	3.47 × 10^−3^	6.68 × 10^−3^	2.48 × 10^−4^	3.74 × 10^−3^	3.74 × 10^−4^	2.67 × 10^−3^	1.47 × 10^−1^	2.67 × 10^−1^	7.48 × 10^−1^
*Penaeus indicus*	3.21 × 10^−3^	4.81 × 10^−3^	3.47 × 10^−4^	4.27 × 10^−3^	1.36 × 10^−3^	2.08 × 10^−2^	9.89 × 10^−1^	3.10	4.09
*Penaeus japonicus*	1.12 × 10^−2^	3.21 × 10^−2^	4.01 × 10^−2^	9.08 × 10^−1^	NA	NA	NA	NA	NA
*Penaeus latisulcatus*	2.03 × 10^−3^	1.98 × 10^−3^	4.27 × 10^−5^	7.48 × 10^−4^	9.89 × 10^−5^	6.41 × 10^−4^	2.94 × 10^−2^	3.21 × 10^−2^	7.75 × 10^−2^
*Metapenaeus ensis*	4.01 × 10^−3^	5.61 × 10^−3^	1.23 × 10^−4^	3.21 × 10^−4^	9.35 × 10^−6^	6.68 × 10^−5^	2.94 × 10^−3^	8.02 × 10^−3^	1.23 × 10^−2^
*Metapenaeus affinis*	2.08 × 10^−3^	2.67 × 10^−3^	5.08 × 10^−5^	3.47 × 10^−4^	2.67 × 10^−5^	4.01 × 10^−4^	2.35 × 10^−2^	1.82 × 10^−2^	5.08 × 10^−2^
*Parapenaeopsis sculptilis*	5.61 × 10^−3^	1.63 × 10^−2^	1.23 × 10^−3^	2.67 × 10^−2^	5.34 × 10^−3^	2.08 × 10^−1^	2.55 × 10^1^	4.16 × 10^1^	2.43 × 10^2^
*Metapenaeopsis barbata*	3.74 × 10^−3^	7.48 × 10^−3^	1.18 × 10^−3^	5.08 × 10^−2^	3.21 × 10^−3^	4.27 × 10^−2^	2.48	3.66	1.01 × 10^1^
*Parapenaeospsis hardwickii*	4.81 × 10^−3^	1.87 × 10^−2^	9.08 × 10^−3^	3.21 × 10^−1^	2.67 × 10^−2^	3.21 × 10^−1^	1.92 × 10^1^	1.09 × 10^1^	2.58 × 10^1^
*Metapenaeus brevicornis*	1.52 × 10^−3^	3.47 × 10^−3^	2.38 × 10^−4^	3.47 × 10^−3^	2.40 × 10^−4^	6.68 × 10^−3^	5.88 × 10^−1^	4.01 × 10^−1^	2.00

NA: not available.

## Data Availability

The original contributions presented in this study are included in the article/Supplementary Material. Further inquiries can be directed to the corresponding author.

## Supplementary Materials

The following supporting information can be downloaded at: https://www.mdpi.com/article/10.3390/toxics14030199/s1, Table S1: Component matrix of the principal component analysis; Table S2: Health risk estimation from consumption of cephalopods and crustaceans in Peninsular Malaysia.

## Abbreviations

The following abbreviations are used in this manuscript:

ICP-MS	Inductively Coupled Plasma Mass Spectrometry
PCA	Principal Component Analysis
EWI	Estimated weekly intake
WHO	World Health Organisation
FAO	Food and Agriculture Organization of the United Nations
USEPA	United States Environmental Protection Agency
HQ	Hazard Quotient
HI	Hazard Index
TCR	Target Cancer Risk
PTWI	Provisional Tolerable Weekly Intake
JECFA	Joint FAO/WHO Expert Committee on Food Additives
IRIS	Risk Information System
IQR	Interquartile range
ND	Not detected
