# Supplementary material for: Health Risk Assessment of Heavy Metals Exposure from the Consumption of Cephalopods and Crustaceans in Peninsular Malaysia
Source: Toxics. 2026 Feb 27;14(3):199. doi: 10.3390/toxics14030199 (PMC13030184; doi:10.3390/toxics14030199)
Supplement: Supplementary file 1 [file toxics-14-00199-s001.zip › toxics-4080063-supplementary.pdf]

Supplementary Materials

Table S1. Component matrix of the principal component analysis.

	**Cumulative 96.6%**	
	Component	
	1	2
Sb	.483	.868
Se	.644	.756
Cd	.913	-.174
Sn	.976	-.192
Pb	.981	-.161
Cu	.980	-.125
Zn	.978	-.111
Cr	.985	-.096
Mn	.973	-.079

Table S2. Health risk estimation from consumption of cephalopods and crustaceans in Peninsular Malaysia.

Species	HQ					HI	TCR	
	Sb	Se	Cd	Zn	Mn		Pb	Cr
Sephia esculenta	2.16E-03	3.60E-04	5.47E-04	1.51E-03	4.01E-03	8.58E-03	5.76E-12	1.20E-07
Sepia phuruonis	1.08E-03	2.30E-04	5.18E-04	1.08E-03	1.95E-03	4.86E-03	3.41E-12	7.09E-08
Loligo duvaucelli	1.04E-03	2.16E-04	5.03E-04	1.08E-03	4.67E-03	7.52E-03	3.67E-12	9.40E-08
Loligo uyii	1.22E-03	2.16E-04	1.01E-03	1.01E-03	6.52E-03	9.98E-03	9.69E-12	1.80E-07
Loligo chinensis	7.73E-04	9.92E-05	3.02E-04	3.36E-04	5.65E-04	2.08E-03	2.23E-12	9.55E-09
Loligo sibogae	9.17E-04	1.87E-04	1.01E-03	1.41E-03	4.57E-03	8.10E-03	4.98E-12	9.86E-08
Loligo edulis	1.02E-03	1.73E-04	4.89E-04	2.33E-03	4.06E-03	8.07E-03	7.60E-12	1.05E-07
Cistopus indicus	2.52E-03	4.46E-04	2.37E-03	4.15E-03	2.57E-02	3.52E-02	4.45E-11	3.88E-07
Penaeus merguiensis	1.10E-03	1.32E-04	1.39E-04	5.73E-05	4.44E-04	1.87E-03	1.19E-13	1.01E-08
Penaeus monodon	2.29E-04	1.58E-08	1.82E-06	3.44E-06	2.82E-06	2.37E-04	9.33E-15	6.05E-11
Penaeus semisulcatus	1.19E-03	1.52E-07	2.82E-05	5.57E-05	6.07E-04	1.88E-03	1.87E-13	6.72E-09
Penaeus indicus	1.10E-03	1.09E-07	3.95E-05	3.74E-04	3.32E-03	4.83E-03	6.80E-13	7.80E-08
Penaeus japonicus	3.84E-03	7.29E-07	4.55E-03	NA	NA	NA	NA	NA
Penaeus latisulcatus	6.95E-04	4.49E-08	4.86E-06	1.11E-05	6.29E-05	7.74E-04	4.93E-14	8.07E-10
Metapenaeus ensis	1.37E-03	1.28E-07	1.40E-05	1.11E-06	9.98E-06	1.40E-03	4.67E-15	2.02E-10
Metapenaeus affinis	7.14E-04	6.07E-08	5.77E-06	8.91E-06	4.12E-05	7.70E-04	1.33E-14	4.57E-10
Parapenaeopsis sculptilis	1.92E-03	3.70E-07	1.40E-04	9.64E-03	1.97E-01	2.09E-01	2.67E-12	1.05E-06
Metapenaeopsis barbata	1.28E-03	1.70E-07	1.34E-04	9.41E-04	8.22E-03	1.06E-02	1.60E-12	9.21E-08
Parapenaeospsis hardwickii	1.65E-03	4.25E-07	1.03E-03	7.27E-03	2.10E-02	3.09E-02	1.33E-11	2.74E-07
Metapenaeus brevicornis	5.22E-04	7.89E-08	2.70E-05	2.23E-04	1.63E-03	2.40E-03	1.20E-13	1.01E-08

HQ: hazard quotient. HI: hazard index. TCR: target cancer risk. NA: not available.
